# Mutations in mitochondrial ribosomal protein MRPL12 leads to growth retardation, neurological deterioration and mitochondrial translation deficiency^☆^

**DOI:** 10.1016/j.bbadis.2013.04.014

**Published:** 2013-08

**Authors:** Valérie Serre, Agata Rozanska, Marine Beinat, Dominique Chretien, Nathalie Boddaert, Arnold Munnich, Agnès Rötig, Zofia M. Chrzanowska-Lightowlers

**Affiliations:** a Université Paris Descartes-Sorbonne Paris Cité, Institut Imagine and INSERM U781, Hôpital Necker-Enfants Malades, 149 rue de Sèvres, 75015 Paris, France; b Department of Pediatrics, Hôpital Necker-Enfants-Malades, 149 rue de Sèvres, 75015 Paris, France; c Wellcome Trust Centre for Mitochondrial Research, Institute for Ageing and Health, Newcastle University, Medical School, Framlington Place, Newcastle upon Tyne, NE2 4HH, United Kingdom

**Keywords:** MRP, mitoribosomal protein, OXPHOS, oxidative phosphorylation, COX, cytochrome *c* oxidase, POLRMT, mitochondrial RNA polymerase, Mitochondria, Mitoribosome, Protein synthesis, Disease, OXPHOS defect

## Abstract

Multiple respiratory chain deficiencies represent a common cause of mitochondrial diseases and are associated with a wide range of clinical symptoms. We report a subject, born to consanguineous parents, with growth retardation and neurological deterioration. Multiple respiratory chain deficiency was found in muscle and fibroblasts of the subject as well as abnormal assembly of complexes I and IV. A microsatellite genotyping of the family members detected only one region of homozygosity on chromosome 17q24.2–q25.3 in which we focused our attention to genes involved in mitochondrial translation. We sequenced *MRPL12*, encoding the mitochondrial ribosomal protein L12 and identified a c.542C>T transition in exon 5 changing a highly conserved alanine into a valine (p.Ala181Val). This mutation resulted in a decreased steady-state level of MRPL12 protein, with altered integration into the large ribosomal subunit. Moreover, an overall mitochondrial translation defect was observed in the subject's fibroblasts with a significant reduction of synthesis of COXI, COXII and COXIII subunits. Modeling of MRPL12 shows Ala181 positioned in a helix potentially involved in an interface of interaction suggesting that the p.Ala181Val change might be predicted to alter interactions with the elongation factors. These results contrast with the eubacterial orthologues of human MRPL12, where L7/L12 proteins do not appear to have a selective effect on translation. Therefore, analysis of the mutated version found in the subject presented here suggests that the mammalian protein does not function in an entirely analogous manner to the eubacterial L7/L12 equivalent.

## Highlights

• MRPL12 function is not entirely analogous to the eubacterial L7/L12 equivalent.
• Mutations in MRPL12 cause translation defects.
• Mutations in apparently universal translation factors can affect different OXPHOS complexes.

## 1 Introduction

The mitochondrial machinery responsible for oxidative phosphorylation (OXPHOS) comprises five enzyme complexes containing approximately 80 proteins of which only 13 are encoded by the mitochondrial genome (mtDNA) [1]. OXPHOS deficiencies affecting a single or multiple complexes can result from mutations in either mitochondrial or nuclear genes and are associated with a variety of disease mechanisms [2,3]. With the advent of Next Generation Sequencing there is an increasing number of pathogenic mutations being identified that are not solely restricted to the 80 genes encoding OXPHOS components, thus highlighting the importance of mechanisms impacting on mitochondrial gene expression [4,5]. Combined OXPHOS deficiencies can arise from alterations in mtDNA, its maintenance [6], cardiolipin levels [7,8], or where none of these are affected, from direct defects in synthesis of mitochondrially encoded proteins [9]. This last group constitutes a heterogeneous mix of patients suffering from a wide range of clinical symptoms making clinical diagnosis difficult [10]. Genetic diagnosis is yet more elusive in children with mitochondrial disease where unidentified nuclear mutations account for the majority of cases [11]. This diagnostic problem is compounded by our relatively poor understanding of the complex molecular machinery that drives translation in mitochondria. This machinery comprises over a hundred proteins [12], all of which are putative candidate genes for translation deficiencies in human. Indeed, translation deficiencies represent a growing cause of multiple OXPHOS deficiencies with several published pathogenic mutations in genes related to the intra-organellar protein synthesis. Although many mutations associated with impaired mitochondrial translation currently map to tRNA genes [13] and a few ribosomal RNA (rRNA) [14], the list of nuclear gene mutations is steadily growing as mutations in genes encoding mitochondrial translation factors such as *GFM1* (OMIM: 606639) [15,16], *TSFM* (OMIM: 604723) [17] and *TUFM* (OMIM: 602389) [18]; mitochondrial aminoacyl-tRNA synthetases (RARS2 (OMIM: 611524) [19], DARS2 (OMIM: 610956) [20], YARS2 (OMIM: 610957) [21], SARS2 (OMIM: 612804) [22], HARS2 (OMIM: 600783) [23], AARS2 (OMIM: 612035) [24], MARS2 (OMIM: 609728) [25], EARS2 (OMIM: 612799) [26]), FARS2 (OMIM: 611592) [27]; tRNA-modifying enzymes (PUS1 (OMIM: 608109) [28], TRMU (OMIM: 610230) [29], MTO1 (OMIM: 614667) [30]); other factors (C12orf65 (OMIM: 613541) [31], TACO1 (OMIM: 612958) [32], LRPPRC (OMIM: 607544) [33], C12orf62 (OMIM: 614478) [34]) and mitochondrial ribosomal proteins (MRPS16 (OMIM: 609204) [35], MRPS22 (OMIM: 605810) [36], MRPL3 (OMIM: 607118) [5]) have been successively reported (reviewed in Ref. [14]). Relatively few cases of OXPHOS deficiencies associated with mutations in mitochondrial ribosomal proteins (MRPs) have been described so far. *MRPS16* mutations have been described in only one family with agenesis of corpus callosum and dysmorphism. *MRPS22* mutations lead to cardiomyopathy, hypotonia and tubulopathy in a first family and Cornelia de Lange-like dysmorphic features, brain abnormalities and hypertrophic cardiomyopathy in another family. Finally, we recently identified *MRPL3* mutations in four siblings of the same family presenting cardiomyopathy and psychomotor retardation. Since the mammalian mitoribosome (55S) is ~ 2 megadalton machine consisting of approximately 80 components that make up the 28S small (SSU) and 39S large subunit (LSU), it is likely that more pathogenic mutations in the constituent polypeptides will be uncovered. One of the substantial differences between the mammalian mitoribosome and those of eubacteria (70S) or the eukaryotic cytosol (80S) is the reversal in the protein to rRNA ratio. The 70S and 80S particles contain ~ 70% rRNA, whilst human mitoribosomes contain ~ 70% protein. This change in the ratio represents both an acquisition of new MRPs as well as loss of bacterial orthologues [37,38]. MRPL12 does have a bacterial orthologue, which through its interactions with translation factors is important in protein synthesis regulating both speed and accuracy [39–41].

Here we investigate the genetic basis of disease in a subject born to consanguineous parents, who initially presented with growth retardation and then neurological distress, with evidence of compromised mitochondrial protein synthesis. We have identified the causative mutation to be in *MRPL12*, encoding a protein of the large subunit of mitochondrial ribosome. This is an important finding indicating that the function and consequence of dysfunction cannot automatically be extrapolated from an apparent orthologue. We show that proteins involved in mitochondrial translation, even close orthologues as submitted here, can defy predictions. Moreover, mutations in such genes that should affect all mitochondrially encoded gene products can exert respiratory chain complex specific defects.

## 2 Materials and methods

### 2.1 Analysis of oxidative phosphorylation activities

Spectrophotometric assays of respiratory chain and complex V enzymes were carried out as previously described [42]. Mitochondrial suspension from cultured skin fibroblasts was obtained after suspending 50 μl of frozen cells in 1 ml of mitochondria extraction medium (20 mM Tris–HCl (pH 7.2), 250 mM sucrose, 2 mM EGTA, 40 mM KCl, 1 mg/ml BSA) supplemented with 0.01% (w/v) digitonin and 10% Percoll (v/v). After 10 min at 4 °C, the sample was pelleted, washed in extraction medium and pelleted before resuspension in 30 μl of extraction medium for respiratory chain enzyme measurements.

### 2.2 Microsatellite genotyping and mutation screening

A genome-wide search for homozygosity was undertaken with 382 pairs of fluorescent oligonucleotides from the Genescan Linkage Mapping Set, version II (Perkin-Elmer) under conditions recommended by the manufacturer. Amplified fragments were electrophoresed and analyzed with an automatic sequencer (ABI 377). The polymorphic markers had an average spacing of 10 cM throughout the genome.

Genes encoding mitochondrial proteins were selected in Mitocarta [43]. The exons and exon–intron boundaries of the *MRPL12* gene were amplified using specific primers (sequences available on request) with initial denaturation at 96 °C — 5 min, followed by 30 cycles of 96 °C — 30 s, 55 °C — 30 s, 72 °C — 30 s, and a last extension at 72 °C for 10 min. Amplification products were purified by ExoSapIT (Amersham, Buckinghamshire, UK) and directly sequenced using the PRISM Ready Reaction Sequencing Kit (Perkin-Elmer, Oak Brook, IL) on an automatic sequencer (ABI 3130xl; PE Applied Biosystems, Foster City, CA).

### 2.3 Cell culture

Human skin fibroblasts were cultured in DMEM medium (Dulbecco's modified Eagle's medium, Gibco) supplemented with 10% (v/v) fetal calf serum (FCS), 2 mM l-glutamine, 50 μg/ml uridine, 110 μg/ml pyruvate, 10,000 U/ml penicillin G and 10,000 μg/ml streptomycin.

### 2.4 Protein analysis

For blue native-polyacrylamide gel electrophoresis (BN-PAGE), mitochondria and OXPHOS complexes were isolated as described [44]. Solubilized OXPHOS proteins (20 μg) were loaded on a 4–16% (w/v) polyacrylamide non-denaturing gradient gel (Invitrogen). SDS–PAGE analysis was performed on either solubilized mitochondrial proteins (40 μg) or cell lysate (50 μg) extracted from cultured skin fibroblasts. After electrophoresis, gels were transferred to a PVDF membrane (GE-Healthcare) and processed for immunoblotting.

### 2.5 Metabolic labelling of mitochondrial translation products

*In vitro* labeling of mitochondrial translation products was a modification from Chomyn et al. [45]. Essentially, cultured skin fibroblasts were preincubated in methionine/cysteine-free DMEM (2 × 10 min) followed by a 10 min in the presence of emetine (100 μg/ml). Radiolabel (125 μCi/ml EasyTag™ express^35^S protein labelling mix — NEG772002MC, PerkinElmer) was then added for 1 h at 37 °C and chased for 1 h. Cells were harvested in cold 1 mM EDTA/PBS, washed 3 times in cold PBS and the pellet resuspended in 30 μl PBS containing 1 × EDTA free protease inhibitors (Roche) and 1 mM PMSF. Samples were treated with 2 × dissociation buffer (20% (v/v) glycerol, 4% (w/v) SDS, 250 mM Tris–HCl pH 6.8, 100 mM DTT) and 12 U Benzonase nuclease (Novagen) for 1 h and separated on a 15% (w/v) SDS–PAGE. The gel was fixed overnight (3% (v/v) glycerol, 10% (v/v) acetic acid, 30% (v/v) methanol) and vacuum dried (60 °C, 2 h). Radiolabelled proteins were visualized by PhosphorImage and analyzed with Image-Quant software (Molecular Dynamics, GE Healthcare).

### 2.6 Homology modeling of the human MRPL12 protein

The three dimensional structure of the human MRPL12 (residues 64 to 198) was modeled by comparative protein modeling and energy minimization, using the Swiss-Model program (http://swissmodel.expasy.org/) in the automated mode. The 2 Å coordinate set for the ribosomal protein L12 from *Thermotoga maritima* (PDB code: 1dd3) was used as a template for modeling the human MRPL12 protein. Swiss-Pdb Viewer 3.7 (http://www.expasy.org/spdbv) was used to analyze the structural insight into MRPL12 mutation and visualize the structures.

### 2.7 Cell lysates, Westerns and isokinetic sucrose gradients

Cell lysates were prepared from fibroblasts by addition of cold lysis buffer (50 mM Tris–HCl pH 7.5, 130 mM NaCl, 2 mM MgCl_2_, 1 mM PMSF, 1% (v/v) NP-40) to cell pellets, which were vortexed for 30 s, centrifuged at 600 *g* for 2 min (4 °C) to remove nuclei and the supernatant retained. These were used for standard westerns as described [46]. Mitochondria were prepared as above and lyzed in 50 mM Tris–HCl pH 7.4, 150 mM NaCl, 1 mM EDTA, 1% Triton X-100, protease inhibitor mix (EDTA free, Roche), 1 mM PMSF, 10 mM MgCl_2_. Mitochondrial lysates (0.3 mg) were loaded on a isokinetic sucrose gradient (1 ml 10–30% (v/v)) in 50 mM Tris–HCl (pH 7.2), 10 mM Mg(OAc)_2_, 40 mM NH_4_Cl, 0.1 M KCl, 1 mM PMSF, 50 μg/ml chloramphenicol), and centrifuged for 2 h 15 min at 100,000 *g* at 4 °C. Fractions (100 μl) were collected and 10 μl aliquots were analyzed directly by western blotting [47].

Immunodetection was performed using the following primary antibodies: anti-CI-Grim19, CII-SDHA 70 kDa, CIII-core2, CIV-COXI, CIV-COXII, CV-subunit β, cyt *c*, NDUFB8 (mouse monoclonal antibodies, MitoSciences); anti-MRPL3 goat polyclonal, MRPS18B rabbit polyclonal, MRPS25 rabbit polyclonal and ICT1 rabbit polyclonal antibodies (Protein Tech Group, Inc., Chicago); anti-DAP3 mouse monoclonal, POLRMT rabbit polyclonal (Abcam); and anti-Porin, mouse monoclonal antibodies (Invitrogen). Anti-MRPL12 rabbit polyclonal antibody was custom made (Eurogentec). Secondary antibody detection was performed using peroxidase-conjugated anti-rabbit, anti-goat or anti-mouse IgG (Abcam or Dako). The signal was generated using ECL + (Pierce, Rockford, USA) and visualised by phosphorimaging and analyzed by ImageQuant software.

### 2.8 RNA and Northern blotting

RNA was isolated from fibroblasts using Trizol following manufacturer's protocol (Invitrogen). Northern blots were performed as described [48]. Briefly, aliquots of RNA (5 μg) were electrophoresed through 1.2% (w/v) agarose under denaturing conditions and transferred to GenescreenPlus membrane (NEN duPont) following the manufacturer's protocol. Radiolabelled probes were generated using random hexamers on PCR-generated templates corresponding to internal regions of the relevant genes.

## 3 Results and discussion

### 3.1 Clinical report

The subject, a boy, was born to first cousin Roma/Gypsy parents by cesarean section, at 41 weeks of pregnancy with severe general hypotrophy (birth weight 2250 g, height 48 cm, Occipitofrontal Circumference 34 cm). His clinical examination at birth was normal and initial investigations failed to identify the cause of his severe hypotrophy (normal blood caryotype, heart ultrasound, bone age, CT scan and metabolic workup). He failed to thrive thereafter and was repeatedly admitted in the first 12 months for gradual worsening of his condition (weight and height: − 4SD, OFC: − 2SD). Clinical examination at 10 months showed severe denutrition, muscle weakness but detectable deep tendon reflexes and no major trunk hypotonia. He started walking with aid at 12 months. Basal growth hormone (GH) levels in plasma were normal (0.5 ng/ml) but plasma IGF1 was very low (0.07 U/ml, normal 0.2). Stimulation by ornithine triggered an adequate elevation of plasma GH with correction of plasma IGF1 (GH: 46.9 ng/ml, IGF1: 0.2 U/ml, and 0.45 U/ml following GH administration). These results ruled out a dwarfism of endocrine origin. Yet, high plasma lactate (3.5 to 4.4 mmol/l, normal below 2.2) prompted skeletal muscle, liver and skin biopsies that revealed a multiple respiratory chain enzyme deficiency in muscle and liver homogenate and cultured skin fibroblasts (Table 1) [42].

He had a first episode of generalized tonic seizure aged 2 years and his neurological condition rapidly worsened following an episode of acute fever (40 °C) caused by a respiratory infection. At that age, he had severe denutrition, flat weight curve, no weight and height gain (− 5 SD) and a mildly enlarged liver. Neurological examination revealed overt psychomotor retardation, severe trunk hypotonia, inability to sit and stand unaided and no speech. Intermittent horizontal nystagmus, with cerebellar ataxia and tremor were noted. He had a mild facial dysmorphism, with round face, epicanthic folds, arched palate, short neck, low-set ears and a unique bilateral median palmar crease. Brain MRI showed T1 hyposignal and T2 hypersignal of white matter and basal ganglia. Rapid aggravation of respiratory conditions required endotracheal intubation and assisted ventilation. Plasma lactates rose to 3.3–4.2 mmol/l, he gradually developed abnormal limb movements then fell into a deep coma, and died following a cardiac arrest.

During the next pregnancy, the mother expected dizygotic twin fetuses. A prenatal diagnosis based on assessment of respiratory chain enzyme activities in cultured skin fibroblasts on amniotic fluid was offered. Hemoglobin contamination of the samples rendered the very low respiratory chain activities; despite this the activity ratios clearly showed a severe complex IV deficiency in cells from the fetus twins leading to termination of the second pregnancy. The same test was normal in the third pregnancy and a normal baby girl was born, now 14 years old (Table 1).

### 3.2 Blue native-PAGE analysis

**Fig. 1:**
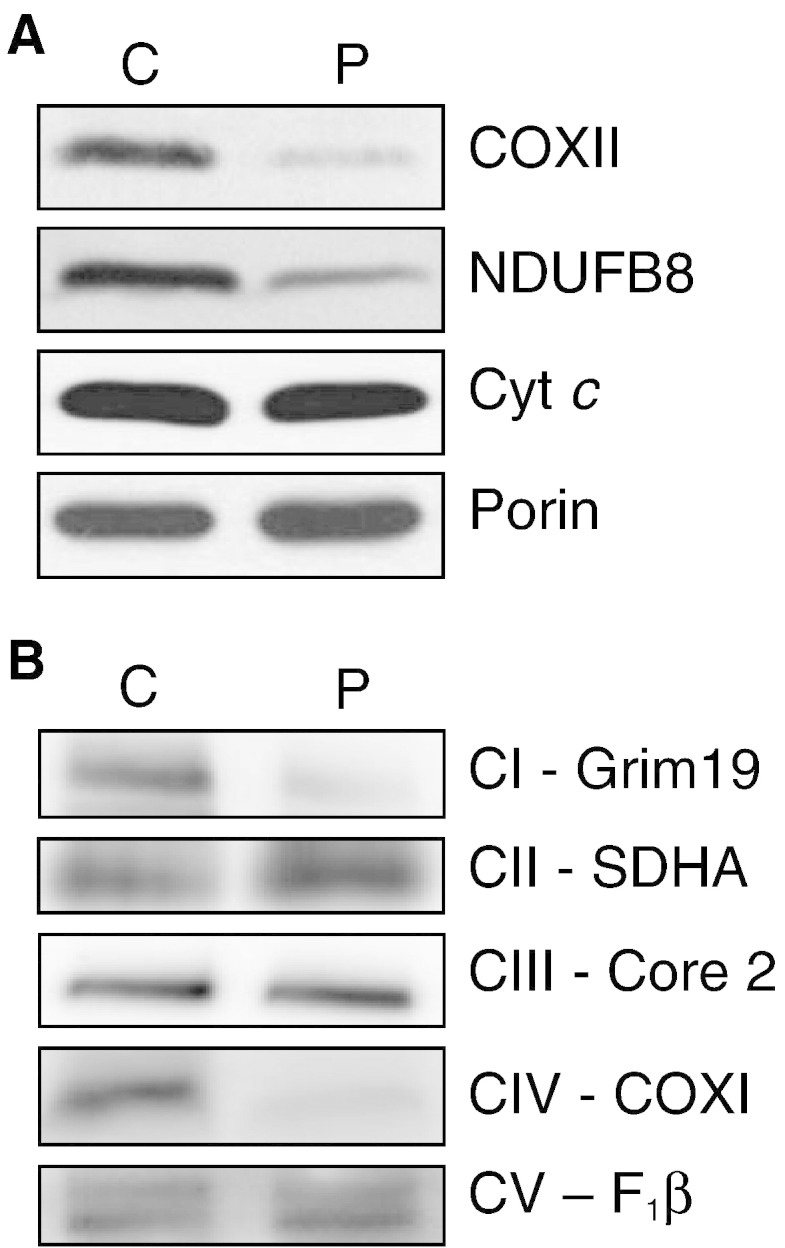
Steady state levels of the oxidative phosphorylation components and complexes. A. Western blot analysis was performed on cytoplasmic extracts (40 μg) of cultured patient (P) and control (C) fibroblasts to determine steady state levels of COXII (MitoSciences). In the absence of other antibodies to mitochondrially encoded proteins, NDUFB8 (MitoSciences) was monitored as a marker for Complex I. Levels of cytochrome *c* (MitoSciences) and porin (Invitrogen) were determined as controls for the respiratory chain and mitochondrial mass respectively. B. BN-PAGE of mitochondria prepared from cultured skin fibroblasts of patient (P) and control (C) was analyzed by western using antibodies (all MitoSciences) directed against GRIM19, SDHA 70 kDa, Core 2, COXI and F_1_β subunit to identify steady state levels of complexes I, II, III, IV and V respectively.

Whilst skin fibroblasts were propagated for enzyme analysis it was clear that those from the subject harboring the mutation had a reduced doubling time on glucose medium compared to control lines (data available on request). Cell extracts were subsequently prepared and SDS–PAGE/western blot analysis revealed reduced steady-state level of COXII subunit, consistent with the decreased CIV activity (Fig. 1A). Moreover, we also observed a decreased amount of nuclear encoded protein NDUFB8 (Fig. 1A), which joins late in complex I assembly [44] and is consistent with the low CI activity in the biopsies. These decreases were consistent with the BN-PAGE data from fibroblast mitoplasts (Fig. 1B, method as described [49]). Both complexes I and IV, detected by anti-GRIM19 antibody or mitochondrially-encoded COXI, were severely decreased in the subject, which in contrast exhibited control levels of complex II (70 kDa SDHA subunit) and complex III (Core 2 subunit) (Fig. 1B). No partial complexes were observed.

### 3.3 Identifying the causative mutation by Microsatellite genotyping and mutation screening

**Fig. 2:**
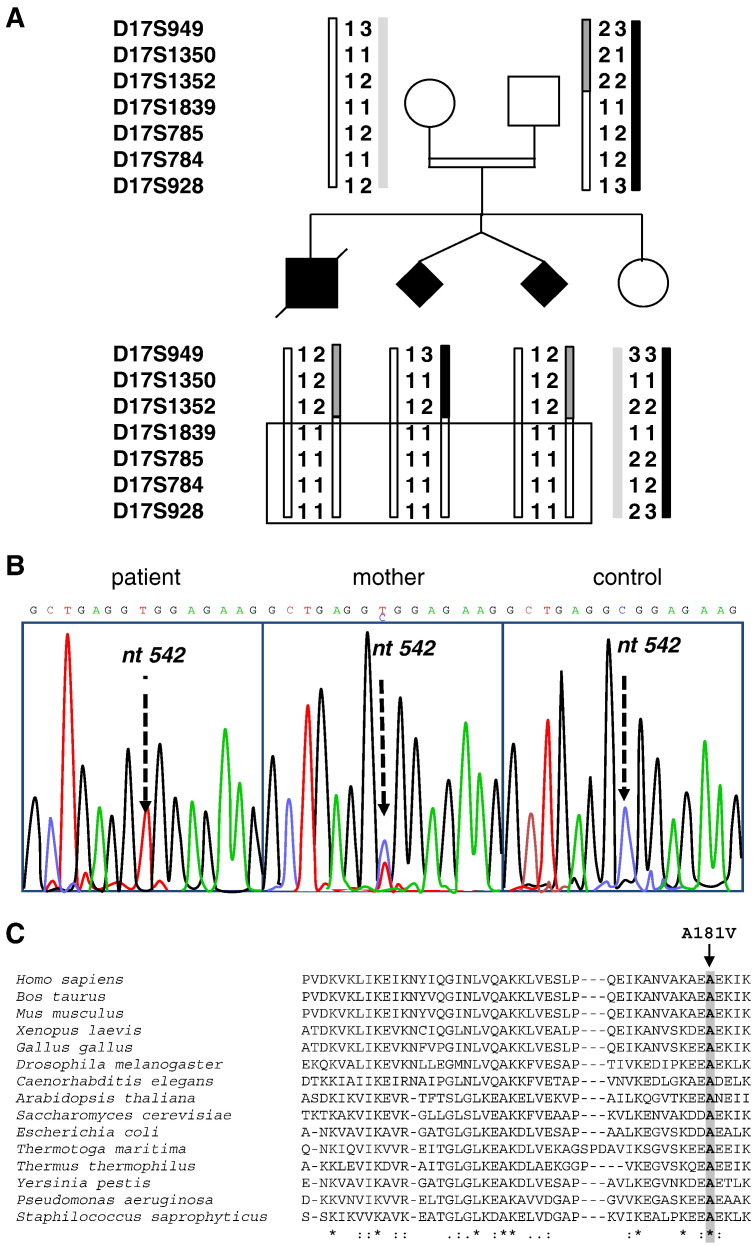
Microsatellite genotyping and mutation screening. A. Pedigree and haplotypes of the family are given (top to bottom) for loci D17S949, D17S1350, D17S1352, D17S1839, D17S785, D17S784 and D17S928. B. Sequence analysis of *MRPL12* in patient subject (left panel), mother (centre panel) and a control (right panel). The arrow indicates the position of the mutation. Amplification products were sequenced using the PRISM Ready Reaction Sequencing Kit (Perkin-Elmer, Oak Brook, IL) on an automatic sequencer (ABI 3130xl; PE Applied Biosystems, Foster City, CA). **C**. Amino acid alignment of MRPL12 (*H. sapiens* to *S. cerevisiae*) and L7/L12 (*E. coli* to *S. saprophyticus*) proteins. The arrow indicates the conserved amino acid that the *MRPL12* mutation changes from an alanine to a valine. Below * designates identity, whilst and: indicate increasing levels of similarity.

In order to identify the causative mutation a genome-wide search for homozygosity was undertaken using 382 polymorphic microsatellite markers (Genescan Linkage Mapping Set, version II (Perkin-Elmer). We obtained evidence for homozygosity in all affected individuals at loci D17S785, D17S784, and D17S928 on chromosome 17q24.2–q25.3 (Fig. 2A). No other homozygous region was found with other markers. This 32.7 cM region was then refined by additional microsatellite markers reducing the critical region to the 25 cM interval, defined by loci D17S1352 and D17S928. This corresponds to a 8.4 Mb physical region containing more than 170 genes, 10 of which encode mitochondrial proteins or proteins predicted to be mitochondrially targeted (*FDXR* (MIM 103270), *ICT1* (MIM 603000), *ATP5H* (MIM 607196), *MRPS7* (MIM 611974), *SLC25A19* (MIM 606521), *MRPL38* (MIM 611844), *PGS1* (NM_024419), *MRPL12* (MIM 602375), *SLC25A10* (MIM 606794), *FASN* (MIM 600212)). Considering the multiple RC deficiency observed in patient muscle, liver and fibroblasts and the abnormal BN-PAGE pattern reminiscent of translation deficiencies, we focused on genes involved in mitochondrial translation, excluding mutations in *MRPS7*, *MRPL38* and *ICT1* by direct sequencing.

Sequencing of *MRPL12* exons and exon–intron boundaries on genomic DNA (primer sequences available on request) from the affected child identified a homozygous c.542C > T transition in exon 5 (RefSeq accession number NM_002949.3, Fig. 2B). This mutation changed a highly conserved alanine into a valine (p.Ala181Val, Fig. 2C) and was predicted by Polyphen2 (http://genetics.bwh.harvard.edu/pph2/) and SIFT (http://sift.jcvi.org/) software to be “probably damaging” and “deleterious” respectively. The parents were heterozygous for the mutation, both twin fetuses were homozygous and the healthy girl was wild-type homozygous. This mutation was absent from 100 controls of the same ethnic origin and from all SNP databases. Further, no additional *MRPL12* mutation could be identified in two other unrelated subjects with similar clinical presentation and biochemical defect.

To demonstrate the deleterious nature of the p.Ala181Val MRPL12 substitution we used, overexpression of wild-type or mutant human *MRPL12* cDNA in the SV40-immortalized fibroblasts but rather recapitulating the respiratory phenotype of the patient fibroblasts, this was found to be lethal. Cells stopped growing, became polynucleated and progressively died.

### 3.4 In silico analysis of the putative impact of A181V substitution

**Fig. 3:**
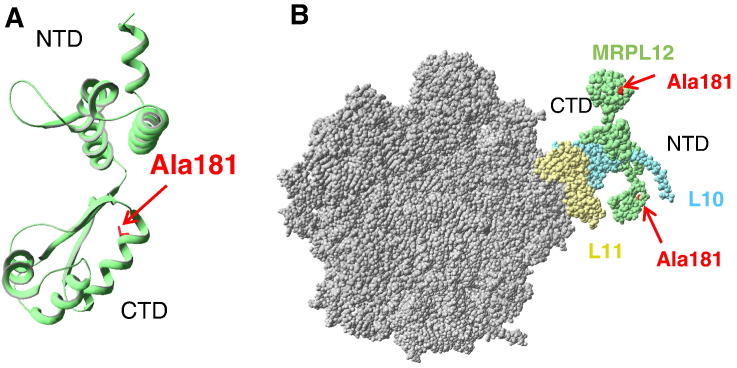
MRPL12 and its position in the ribosome. A. The three dimensional structure of the human MRPL12 (residues 64 to 198) was modeled by comparative protein modeling and energy minimization, using the Swiss-Model program in the automated mode. The 2 Å coordinate set for the ribosomal protein L12 from *Thermotoga maritima* (PDB code: 1dd3) was used as a template for modeling the human MRPL12 protein. Swiss-Pdb Viewer 3.7 (http://www.expasy.org/spdbv) was used to analyze the structural insight into MRPL12 mutation and visualize the structures. The A181 residue shown (in red) is localized in a helix that is likely to be at the interface of an interaction with translation factors. B. Potential MRPL12 interactions within the ribosomal L7/L12 stalk. Swiss-Pdb Viewer 3.7 (http://www.expasy.org/spdbv) was used to superimpose MRPL12 on the *Thermus thermophilus* 70S ribosome (PDB code: 2WRL) without L7/L12 stalk proteins. MRPL12, L10 and L11 are shown in green, blue and yellow respectively. The Ala181 residue (in red) is located in the MRPL12 CTD. The p.Ala181Val change has a high probability of altering interactions of MRPL12 with elongation factors and might be predicted to affect initial binding, decreasing both rate and accuracy of mitochondrial translation. NTD: N-terminal domain; CTD: C-terminal domain.

Human MRPL12 is 27.5% identical in amino acid sequence to the previously crystallized *T. maritima* L12 ribosomal protein. The C-terminal domain (CTD) (107 to end in *Escherichia coli*) is conserved in evolution [50] and is required for initial binding and GTPase activation for both EF-Tu and EF-G. Indeed, both EF-Tu and EF-G have greatly diminished GTPase activity on ribosomes lacking the CTD of L12 [51,52]. Superimposition of MRPL12 with the *Thermus thermophilus* 70S ribosome (PDB code: 2WRL) lacking L7/L12 stalk proteins shows that MRPL12 Ala181 is located within this highly conserved region (Fig. 3A). Moreover, modeling of MRPL12 shows Ala181 positioned in a helix potentially involved in translation factor interactions (Fig. 3A/B). Bacterial L7/L12 CTDs also contain a number of strictly conserved residues that are involved in the initial contact with elongation factors [52,53] and crucial for translation [54]. Alanine is one of the best helix forming residues and substitutions can therefore have profound energetic effects by perturbing packing interactions or tertiary contacts [55]. Thus, the p.Ala181Val change might be predicted to alter interactions with the elongation factors, and since MRPL7/12 bound to elongation factors is predicted to have a higher affinity for the ribosome [54], the mutation may in turn affect both rate and accuracy of mitochondrial translation.

### 3.5 Ribosome assembly

**Fig. 4:**
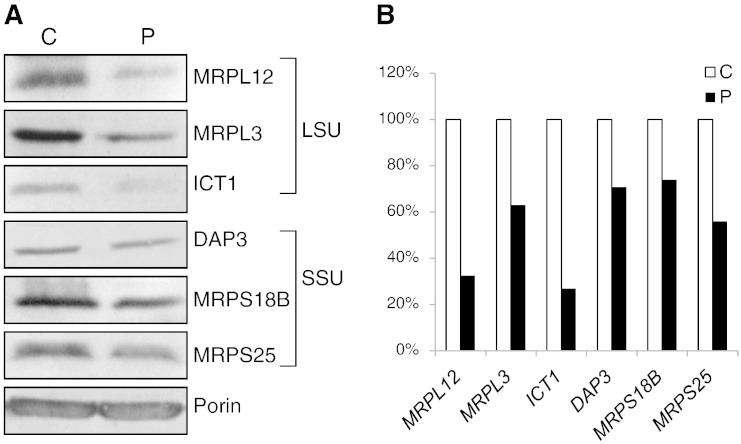
Analysis of mitoribosomal components. A. Cell lysates (50 μg) from patient (P) and control (C) fibroblasts were separated by SDS–PAGE followed by western to decorate various components. Polypeptides of the large ribosomal subunit (LSU) included MRPL12 (Eurogentec), MRPL3 (PTG labs), ICT1 (PTG labs) and small ribosomal subunit (SSU) included DAP3 (Abcam), MRPS18B (PTG labs) and MRPS25 (PTG labs). Porin (Invitrogen) was used as a loading control. B. Densitometric values from 3 independent experiments as described in panel A represent the levels of MRPL12, MRPL3, ICT1, DAP3, MRPS18B and MRPS25 in subject compared to control fibroblasts.

The steady state level of MRPL12 in the subject's fibroblasts was reduced to 30% of control value (Fig. 4A and B). The mt-LSU protein ICT1 was also decreased (~ 30% of control values) as was MRPL3 (by 37%) suggesting that a consequence of the MRPL12 mutation is a global defect in assembly of the large ribosomal subunit (mt-LSU). In order to estimate the effect of the MRPL12 mutation on assembly of the whole ribosome, we also tested three proteins of the small ribosomal subunit (SSU), MRPS18B, MRPS25 and DAP3. These were modestly decreased with levels of ~ 60–80% of control (Fig. 4A and B). Correspondingly, 16S and 12S rRNA levels were decreased by 35% and 22% respectively (Fig. 7A). Since porin indicated that there was no compensatory mitochondrial biogenesis and staining of the mitochondrial network with tetramethylrhodamine methyl ester showed no significant alteration in amount or distribution of mitochondria (AR and ZCL unpublished observation) we conclude that the *MRPL12* mutation destabilizes the protein resulting in less mt-LSU and to a lesser extent of the small subunit.

**Fig. 5:**
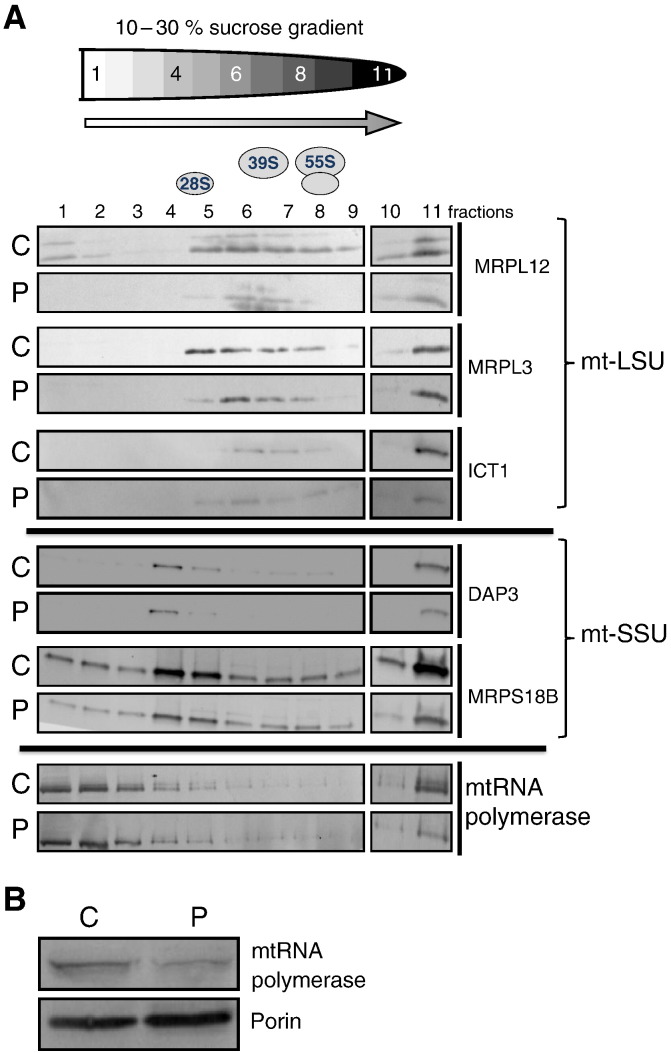
Isokinetic gradient analysis of mitochondrial lysates. A. Mitochondrial lysates were prepared from patient (P) and control (C) fibroblasts and 300 μg of each was separated through 10–30% (w/v) isokinetic sucrose gradients. The levels of 28S small subunit (SSU) and 39S large subunit (LSU) proteins were determined by western blot analysis. 55S corresponds to the assembled mitoribosome. The steady state level (panel B. porin as loading control) and distribution (panel A) of POLRMT was also examined. Antibodies as in previous legends except POLRMT (Abcam).

In order to determine whether the MRPL12 mutation also induced changes in composition and assembly of the mitochondrial ribosomal large and small subunits, mitochondrial lysates from cultured fibroblasts (subject and control) were fractionated on isokinetic sucrose gradients (10–30%, as in Ref. [47]). If assembly of either the large subunit or the entire ribosome was affected then the distribution of individual ribosomal proteins would change within the gradient profile. On analysis MRPL12 from the patient was substantially decreased in all fractions but detectable in the fractions consistent with mt-LSU; however it was noticeably absent from the free pool (fractions 1 and 2, Fig. 5). This was in contrast to the control that exhibited a pool of free MRPL12, which has been reported to interact with POLRMT [56]. MRPL3 was also slightly reduced in subject cells but remained in fractions consistent with the large subunit. The MRPL12 mutation impacted more modestly on the small ribosomal subunit, with DAP3 apparently unaffected and MRPS18B found in lower amounts only in fractions 4 and 5 but otherwise with similar steady state levels and distribution profile compared to control. Since POLRMT and MRPL12 have been published as interactors, we analyzed both the steady state level and gradient distribution of POLRMT to see if these were affected by the MRPL12 mutation. Overall levels in the subject sample were decreased to 63% of control value (Fig. 5B) but distribution in the gradient appeared largely unaffected with the exception of fraction 11, where levels were lower than control (Fig. 5A bottom panels).

### 3.6 In vitro translation

**Fig. 6:**
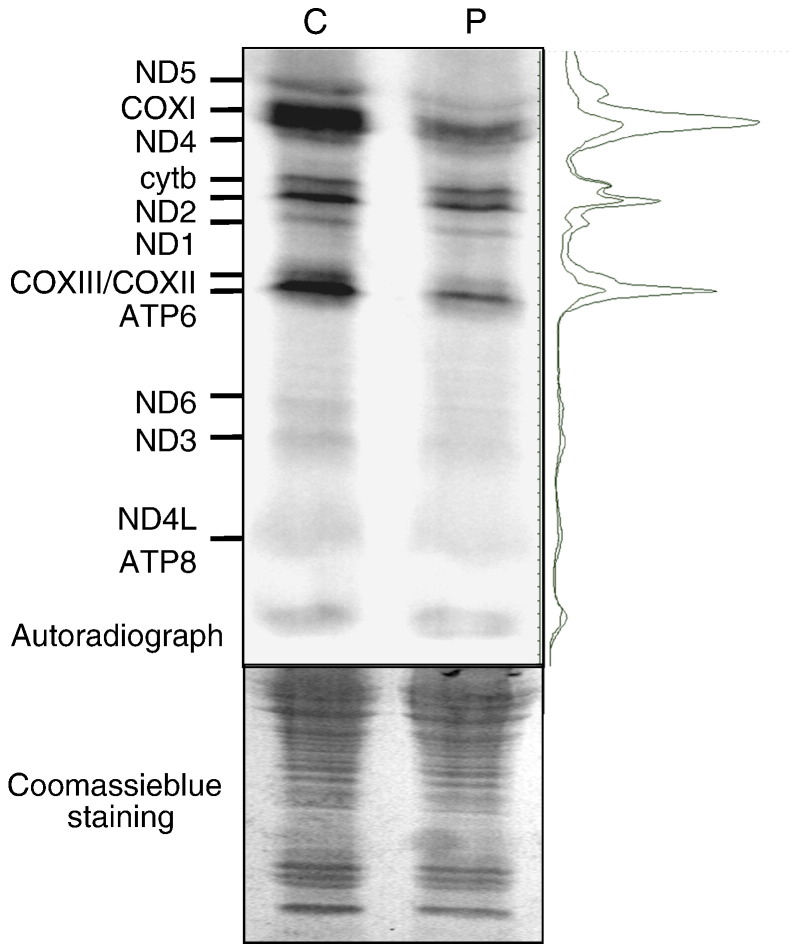
Mitochondrial protein synthesis. *De novo* synthesis of mitochondrial proteins was determined in patient (P) and control (C) fibroblasts under conditions that inhibited cytosolic translation [45]. *In vivo* incorporation of ^35^S-methionine/cysteine into mitochondrially encoded proteins was visualised by separation of cell lysate (50 μg) through SDS–PAGE, exposure of the dried gel to a PhosphorImage screen, followed by Storm and ImageQuant analysis (upper panel). To the right of the gel are the aligned densitometric profiles of the patient (lower trace) and control (upper trace). The gel was subsequently rehydrated and stained with Coomassie blue to confirm equal loading (lower panel).

To identify any effect on global mitochondrial protein synthesis, we studied *de novo* mitochondrial translation in cultured skin fibroblasts, as described in Ref. [45]. Although there was an overall decrease in mitochondrial translation compared to control, densitometric profiles showed that certain polypeptides were more affected than others (Fig. 6). In particular, there was a significant reduction of synthesis of COXI, COXII and COXIII subunits. Consistent with the respiratory chain activities, complex I polypeptides were affected to a lesser extent and cytochrome *b* from complex III appears to be spared. Despite the potential role of MRPL12 in translational accuracy, no aberrant translation product could be detected.

### 3.7 Steady-state level of mitochondrial transcripts

**Fig. 7:**
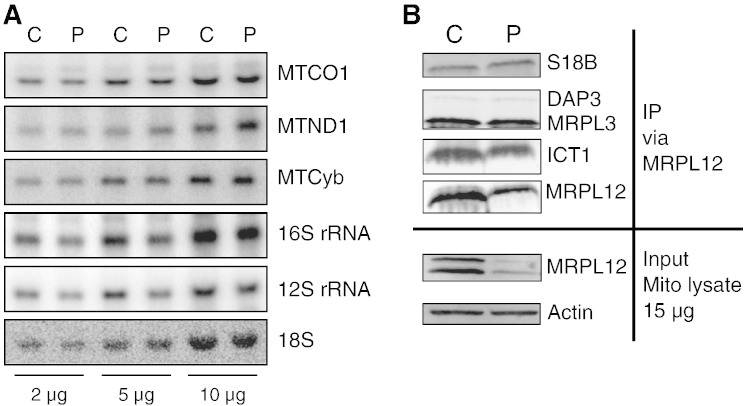
Analysis of mitochondrial transcripts and of MRPs immunoprecipitating with MRPL12. A. Steady-state levels of mitochondrial transcripts from patient (P) and control (C) fibroblasts were analyzed by Northern blot. Signals were normalized against 18S cytosolic rRNA. Three different amounts (2, 5 and 10 μg) of total RNAs were loaded. B. MRPL12 was immunoprecipitated from mitochondrial lysates (835 μg) prepared from patient (P) and control (C) fibroblasts. Recovered MRPL12 and co-immunoprecipitating MRPs were analyzed by western blot (antibodies as previously described).

MRPL12 has been shown to interact with the mitochondrial RNA polymerase (POLRMT) and to stimulate mitochondrial transcription [56,57]. Since the steady-state level of POLRMT in subject fibroblasts was modestly decreased, we analyzed the steady-state level of mitochondrial transcripts to see if these were similarly affected. Northern blots on control and subject fibroblast RNA did not show a decrease that paralleled the reduced levels of POLRMT. In fact there was a slight increase of *MTND1* in subject cells compared to control, whilst *MTCOI* and *MTCYB* transcripts appeared unaffected (Fig. 7A). Conversely, as mentioned earlier, the levels of 16S and 12S rRNA were modestly decreased (Fig. 7A) in a proportion that was consistent with the loss of MRPs with which they would associate. Analysis of the distribution on 10–30% (w/v) sucrose gradients (as in Ref. [58]) of two mt-mRNAs, *MTCOI* and *MTND1*, showed a relatively similar pattern for subject and control but in each case a smaller proportion of the subject transcript sedimented to the final fraction (data available on request). This was also true for the 16S and 12S rRNA. As no major redistribution in the sucrose gradient was observed, it is unlikely that the MRPL12 mutation causes a global defect in assembly of the mitoribosome.

Since the levels of POLRMT were slightly decreased with normal or slightly elevated levels of mt-mRNA, we assessed the stability of transcripts to see if the half-lives were extended to compensate for reduced synthesis in order to maintain normal steady state levels. Mitochondrial transcription was poisoned by addition of low levels of ethidium bromide and RNA prepared at numerous time points thereafter (0–16 h). Densitometry of the subsequent Northern demonstrated that half-lives of *MTCOI* and *MTND1* were extended, the latter more so, consistent with the modest increase in steady state levels (data available on request).

### 3.8 MRPL12 dimerization and interaction with the mitoribosome

MRPL12 is the orthologue of eubacterial L7/L12, where L7 is identical to L12 except that it is N-terminal acetylated. L7/L12 is also phosphorylated, which can affect both conformation and binding to partner ribosomal proteins (reviewed in Ref. [37]). This modification has now been confirmed to be present in mammalian MRPL12 [59]. In eubacteria, association of L7/12 to the large subunit takes place via the L10 protein such that two L7/L12 heterodimers normally associate per LSU [60]. Interestingly these dimers are actively exchanged on the 70S molecule without disruption of the ribosomal particle [54,61]. The dimerization status and number of dimers attached to the human 55S has not been clarified. In order to identify if the stoichiometry of MRPL12 per mt-LSU was altered as a consequence of the mutation, we performed immunoprecipitation (IP) analysis on subject and control fibroblasts using antibodies to MRPL12. Analysis of the immunoprecipitate demonstrated similar levels small subunit polypeptides including DAP3 and MRPS18B in subject and control samples. In contrast, the total amount of MRPL12 was reduced (Fig. 7B). In the patient the IP is restricted to MRPL12 in the large subunit or the fully assembled 55S with the total amount of MRPL12 being reduced as it lacks the “free” population. The densitometric measurements indicate that the patient IP has ~ 49% MRPL12 compared to control, in accordance with the gradient and steady state data. ICT1 appears to be sensitive to the MRPL12 levels and so is reduced in both the IP (~ 58% of control) and in the steady state westerns (Fig. 4). The lower levels of MRPL12 could reflect loss of multimerization but since the region in the bacterial protein involved in multimerization is towards the N-terminus [62], this mutation is unlikely to have an impact on dimer/multimer formation. The translation factor bound dimer has been suggested to have an increased affinity for the ribosome [54,61]. Thus a possible explanation is that the mutation affects translation factor binding, thereby reducing the affinity of the mutant MRPL12 for the ribosome. If this were the case, however, then we would expect an increase in the pool of free MRPL12 whereas the subject exhibits a reduced pool of free MRPL12, which interacts with POLRMT [57]. The immunoprecipitation was performed using an MRPL12 specific antibody and so should contain all free MRPL12, MRPL12 associated with uncomplexed mt-LSU and MRPL12 as part of the fully assembled 55S. Since the levels of small and large subunit proteins appeared to be similar in subject and control, these data suggest that the mt-LSU and 55S assembly are unaffected by the mutation consistent with the gradient data for the protein and RNA components. Thus the reduced levels of mutant MRPL12 in this subject correspond to i) loss of stability, ii) a decrease in the free pool that is believed to interact with the mitochondrial RNA polymerase and iii) reduced translation potentially resulting from decreased interactions with translation factors, but with no detectable increase in aberrant translation products.

In conclusion, we report a mutation in human *MRPL12* that results in growth retardation and neurological distress. It is interesting to note that whereas the eubacterial orthologue is not essential for in vitro translation assays, this *MRPL12* mutation induces a mitochondrial translation defect in human. This lack of predictability between orthologous proteins makes it important to examine and not assume what impact mutant forms or loss of MRPs may have on mitochondrial homeostasis and the resulting clinical manifestation. The data presented here provide another example, amongst a growing list of translation factors, which despite their apparent universal contribution to the synthesis of all mitochondrially encoded proteins, has a selective effect on the different oxidative phosphorylation complexes.

## Figures and Tables

**Table 1:** Respiratory chain activities in muscle mitochondria, liver homogenate and fibroblasts.

	Muscle mitochondria		Liver homogenate		Liver homogenate			Fibroblasts		Amniotic cells			
	P	C	P	C	Twin 1 fetus	Twin 2 fetus	C	P	C	Twin 1 fetus	Twin 2 fetus	3rd pregnancy	C
*Absolute activities (nmol/min/mg prot)*													
CI	–	–	–	–	–	–	–	17	27–44	–	–	–	–
CII	170	75–157	–	–	63	57	74–196	59	33–71	–	–	–	–
CIII	697	494–1004	493	128–217	106	73	70–236	505	318–820	1.44	2.1	16.5	19–21
CIV	374	540–1073	7	131–241	57	32	68–284	90	189–429	0.88	0.77	32	26–36
CV	538	209–454	–	–	–	–	–	100	53–133	–	–	–	–
CI + III	89	113–311	27	35–67	–	–	–	–	–	–	–	–	–
CII + III	443	164–357	365	50–98	37	23	38–104	167	69–146	2.22	1.38	8.7	6.5–8.4
CS	–	–	–	–	65	45	40–104	293	112–264	–	–	–	–

*Activity ratios*													
CIV/CI + III	4.2	3.4 ± 0.8	–	–	–	–	–	–	–	–	–	–	–
CIV/CII + III	0.8	3.1 ± 0.5	0.01	3.0 ± 0.4	1.5	1.39	2.7 ± 0.3	0.5	3.0 ± 0.2	0.61	0.55	3.6	2.1–3.3
CIV/CI	–	–	–	–	–	–	–	5.4	10.1 ± 1.0	–	–	–	–
CIV/CII	–	–	–	–	–	–	–	1.5	6.1 ± 0.7	–	–	–	–
CIV/CIII	0.5	1.5 ± 0.2	0.01	2.0 ± 0.6	0.54	0.4	1.2 ± 0.1	0.2	0.6 ± 0.04	0.39	0.37	1.9	0.7–1.2
CIV/CV	0.7	2.5 ± 0.8	–	–	–	–	–	0.9	3.5 ± 0.4	–	–	–	–

CI–CV, complexes I–V; CS, citrate synthase; P, subject; C, control. Abnormal activity values and ratios are shown in bold. Spectrophotometric assays of respiratory chain and complex V enzymes were carried out as previously described [42]. Haemoglobin contamination of amniotic cells reduced accuracy of the absolute values of RC complex activities but not the ratios.
